# Accelerative effect of topical *Zataria multiflora* essential oil against infected wound model by modulating inflammation, angiogenesis, and collagen biosynthesis

**DOI:** 10.1080/13880209.2020.1861029

**Published:** 2020-12-30

**Authors:** Mohammad Reza Farahpour, Sara Sheikh, Elham Kafshdooz, Ali Sonboli

**Affiliations:** a Department of Clinical Sciences, Faculty of Veterinary Medicine, Urmia Branch, Islamic Azad University, Urmia, Iran; b Department of Basic Sciences, Faculty of Veterinary Medicine, Urmia Branch, Islamic Azad University, Urmia, Iran; c Department of Microbiology, Urmia Branch, Islamic Azad University, Urmia, Iran; d Department of Biology, Medicinal Plants and Drugs Research Institute, Shahid Beheshti University, G.C. Evin, Tehran

**Keywords:** Infected wound healing, inflammatory cytokines, antimicrobial properties, thymol

## Abstract

**Context:**

*Zataria multiflora* Boiss (Lamiaceae) essential oil (ZME) is believed to be a bactericide herbal medicine and might alleviate negative effects of infection.

**Objective:**

This study evaluates the effects of an ointment prepared from ZME (ZMEO) on infected wounds.

**Materials and methods:**

A full-thickness excisional skin wound was surgically created in each mouse and inoculated with 5 × 10^7^ suspension containing *Pseudomonas aeruginosa* and *Staphylococcus aureus*. The BALB/c mice (*n* = 72) were divided into four groups: (1) negative control that received base ointment (NCG), (2) positive control that daily received Mupirocin^®^ (MG), (3) therapeutic ointment containing 2% ZMEO and (4) therapeutic ointment containing 4% ZMEO, for 21 days. Wound contraction, total bacterial count, histopathological parameters, antioxidant activity, qRT-PCR analysis for expression of IL-1β, TNF-α, VEGF, IGF-1, TGF-β, IL-10, and FGF-2 mRNA levels were assessed on days 3, 7, and 14 following the wounding.

**Results:**

Topical administration of ZMEO significantly decreased the total bacterial count and wound area and also expression of IL-1β and TNF-α compared to the control groups (*p* < 0.05) in all days. This could also increase significantly the expression of TGF-β, IL-10 IGF-1, FGF-2, and VEGF, and also angiogenesis, fibroblasts, fibrocytes, epithelialization ratio, and collagen deposition and improve antioxidant status compared to the control group (*p* < 0.05).

**Discussion and conclusion:**

ZMEO accelerated the healing process of infected wounds by shortening the inflammatory factors and increasing proliferative phase. Applying ZMEO only and/or in combination with chemical agents for the treatment of wound healing could be suggested.

## Introduction

Wound infections are important challenges that cause the suffering of patients and economic losses (Vittorazzi et al. 2016; Daemi et al. 2019a; Farghali et al. 2019). Skin is a regenerative organ which acts as a barrier between the body and the external environment (Hassan et al. 2014). A wound is defined as a disturbance in anatomic structure of skin and its functional integrity (Farghali et al. 2019). Skin prevents the penetration of bacteria and fungi that are important challenges that induce mortality and morbidity. *Pseudomonas aeruginosa* and *Staphylococcus aureus* are opportunistic bacteria that induce infection in patients (Cardona and Wilson 2015). The bacteria are commonly detected in the upper and deepest region of wound bed (Serra et al. 2015). Colonization of *S. aureus* and *P. aeruginosa* in the wound site postpones the wound healing process (Farahpour et al. 2020; Khezri et al. 2020). The wound healing process comprises several phases, including coagulation, inflammation, epithelialization, granulation tissue formation, and tissue remodelling (Daemi et al. 2019a, 2019b; Farahpour et al. 2020). Inflammatory phase occurs after activation of inflammatory chemokines (Farahpour et al. 2020). Interleukin-1β (IL-1β) is significantly synthesized 12–24 h after infliction of the wound, and its level returns to basal level after the proliferative stage is completed (Fahey and Doyle 2019). Tumour necrosis factor-α (TNF-α) is significantly synthesized by macrophages and T lymphocytes and its level increases under inflammation and infection conditions (Xue and Falcon 2019). Following the induction of inflammation, proliferative phase occurs in which many genes are involved. Insulin-like growth factor 1 (IGF-1) promotes the production of keratinocyte and fibroblast proliferation and improves the re-epithelialization (Daemi et al. 2019b). Decreased endothelial insulin/IGF-1 signal delays in the wound healing process (Aghdam et al. 2012). Vascular endothelial growth factor (VEGF) induces angiogenesis and stimulates cell migration and proliferation (Farahpour et al. 2020). Fibroblast growth factor-2 (FGF-2) is a protein that participates in the wound healing process (Souto et al. 2020). Interleukin-10 (IL-10) reduces the production of pro-inflammatory cytokine, but tumour growth factor-β (TGF-β) has roles in increasing the proliferative phase improving the proliferation and differentiation of fibroblasts, collagen production, and wound contraction (Khezri et al. 2020). On the other hand, oxidative stress increases under wound disorders and increases the production of reactive oxygen species (ROS) in the site of wound (Ustuner et al. 2019). Applying antibacterial and antioxidant agents shortens the inflammatory phase, promotes the proliferative phase and accelerates the wound healing process (Bardaa et al. 2016; Vittorazzi et al. 2016; Daemi et al. 2019a, 2019b).

*Zataria multiflora* Boiss (Lamiaceae) (ZM) has antibacterial activity against *Pseudomonas* spp. (Barkhori-Mehni et al. 2017). Its antibacterial activity is attributed to its phenolic compounds that destroy the cell wall in bacteria (Barkhori-Mehni et al. 2017). It demonstrates antioxidant properties due to its compounds such as thymol and carvacrol (Mojaddar Langroodi et al. 2019). Seemingly, ZM can accelerate the wound healing process due to its antibacterial and antioxidant features, but no studies have been conducted yet to evaluate the *Zataria multiflora* essential oil (ZME) on the wound healing process. Therefore, this study was conducted to evaluate the effect of an ointment prepared from ZME on the healing process of infected wound model by *S. aureus* and *P. aeruginosa* by assessing wound contraction, total bacterial count, histopathological and immunohistochemical, antioxidant activity, and qRT-PCR analyses.

## Materials and methods

### Materials

*Zataria multiflora* essential oil was purchased from Barij Essence Company (Kashan-Iran) and approved by the same herbarium company (No.1203). Commercial base ointment and mupirocin were prepared from Pars Daruo. Ltd., Tehran, Iran. The commercial base ointment contained 90% soft paraffin, 5% hard paraffin and 5% lanolin. Rodent feeds were prepared from Javaneh Khorasan Company (Khorasan, Iran).

### Essential oil analysis and identification

Gas chromatography (GC) analysis was employed in order to identify the compounds. The components of the essential oil were recognized using calculation of their retention indices under temperature-programmed conditions for *n*-alkanes (C6–C24) and the oil on DB-5 column.

### Animals

Seventy-two BALB/c mice weighing 25 ± 3 g were prepared. The mice had free access to water and pelleted feed for rodents (Javaneh Khorasan Company, Khorasan, Iran). The mice were kept in an ambient temperature of 23 ± 3 °C, at a constant air humidity, and a natural day–night cycle. This study was approved by the Animal Research Committee of the Urmia Islamic Azad University with ethical No. IAU-UB 11044.

### Induction of wound

To induce the wound, the mice were primarily anesthetized by intraperitoneal administration route using 50 mg/kg ketamine and 5 mg/kg xylazine. Following the induction of anaesthesia, dorsal region of each mouse was shaven for surgical actions. A circular wound model was induced in a size of 7 mm using a surgical biopsy punch and per wound was inoculated by an aliquot of 5 × 10^7^ suspension containing *S. aureus* (ATCC 26313) and *P. aeruginosa* (ATCC 27853) in 50 μL phosphate-buffered saline (Farahpour et al. 2020; Khezri et al. 2019). Subsequently, animals were divided into four groups (*n* = 18), as (group I) negative control group (NCG) that was administered commercial base ointment, (group II) positive control group that were treated with Mupirocin^®^ (MG), and (groups III and IV) treated groups with therapeutic ointments containing 2 g and 4 g ZME mixed in NCG (2% and 4% ZMEO), respectively. Concerning the colonization of the bacteria, 24 h after the induction of the wounds, ointments were applied on wounds, once a day. In addition, according to the tissue sampling on days 3, 7, and 14 after the wounding, the animals in each group were divided into three subgroups (*n* = 6).

### Wound area

The rate of wound closure was estimated as reported previously (Nejati et al. 2015; Khezri et al. 2019). In order to calculate the percentage of wound closure rate, a transparent paper was placed on it and it was calculated based on the following formula:

Percentage of wound closure = [(Wound area on Day 0 − Wound area on Day X)/Wound area on Day 0] × 100.

### The investigation of the bacteriological count in the wound area

To calculate the bacteriological count, the granulated tissues were excised and 0.1 g of the sample was crushed, minced, and homogenized in a sterile mortar containing 10 mL of sterile saline. It was the diluted in tubes containing 9 mL of sterile saline, was cultured on plate count agar (Merck KGaA, Darmstadt, Germany), incubated at 37 °C for 48 h, calculated as CFU/g of granulation tissue (Farahpour et al. 2020; Khezri et al. 2019).

### Histological analysis

Concerning the evaluation of the histological parameters, the mice were euthanized by a special CO_2_ device and the granulation tissue were excised in along to 1 to 2 mm from surrounding normal skin. The samples were fixed in neutral-buffered formalin 10%, routinely processed, and paraffin wax was embedded, sectioned at 5 µm, and stained with Masson’s trichrome and then examined under light microscopy (Olympus CX31RBSF attached cameraman) as reported by Farahpour et al. (2018). Cellular infiltration, edoema and collagen deposition were assessed. An image pro-insight software was utilised for evaluating the collagen deposition. Morphometric lens (Olympus, Germany) was used for assessing the epithelial thickness. edoema was assessed as a 5-score as reported previously (Nejati et al. 2015).

### Immunohistochemical staining (IHC) for angiogenesis (CD31)

To assess the IHC, it was conducted as reported by Farahpour et al. (2018). The IHC was conducted based on manufacturer’s protocol (Biocare, Yorba Linda, CA). The tissue sections were rinsed gently in the washing buffer and placed in a buffer bath. A DAB chromogen was employed for assessing the tissue sections and then incubated for 5 min. The sections were then dipped in weak ammonia (0.037 M/L) for 10 times, rinsed with distilled water and cover slipped. Brown stains were considered as positive immunohistochemical.

### Fluorescent staining for collagen

To wash the slides, acetic acid 1% was used for several minutes. The slides were stained using acridine-orange. Phosphate buffer was utilized to remove staining. The slides were the washed in distilled water, mounted with buffer drop and analyzed with a fluorescent microscope. The bunds with red and yellowish red colour were considered as collagen bunds (Farahpour et al. 2018).

### RNA isolation and cDNA synthesis

The wound tissues were isolated as previously reported and RNA was extracted applying a standard TRIZOL procedure as reported by Farahpour et al. (2018), and assessed by spectrophotometer (260 nm and 260/280 = 1.8–2.0). To prepare the cDNA, it was put in a reaction being mixed with 1 µg RNA, oligo (dT) primer (1 µL), 5 × reaction buffer (4 µL), RNAse inhibitor (1 µL), 10 mM dNTP mix (2 µL) and M-MuLV Reverse Transcriptase (1 µL) as described by producer protocol (Fermentas, GmbH, Germany). Time and temperature were as reported by Farahpour et al. (2018). The used primers included IL-1β, forward (5′-AAC AAA CCC TGC AGT GGT TCG-3′) and reverse (5′-AGCTGCTTCAGACACTTGCAC-3′); TGF-β, forward (5-CCAAACGCCGAAGACTTATCC-3′) and reverse (5′-CTTATTACCGATGGGATGGGATAGCCC-3′); IL-10, forward (5′-GAAGCTCCCTCAGCGAGGACA-3′) and reverse (5′-TTGGGCCAGTGAGTGAAAGGG-3′), TNF-α, forward (5′-GAAGCTCCCTCAGCGAGGACA-3′) and reverse (5′-TTGGGCCAGTGAGTGAAAGGG-3′), FGF-2, forward (5′-GGAACCCGGCGGGACACGGAC-3′) and reverse (5′-CCGCTGTGGCAGCTCTTG GGG-3′); VEGF, forward (5′-GCTCCGTAGTAGCCGTGGTCT-3′) and reverse (5′-GGAACCCGGCGGGACACGGAC-3′), and IGF-1, forward (5′-TAGGTGGTTGATGAATGGT-3′) and reverse (5′-GAAAGGGCAGGGCTAAT-3′).

### Antioxidant capacity

To assess the antioxidant capacity, the wound granulation tissue was homogenized in ice-cold KCl (150 mM) and the mixture was then centrifuged at 3000 *g* for 10 min. The supernatant was used to evaluate the total antioxidant capacity (TAC), malondialdehyde (MDA) and total tissue thiol molecules (TTM) content. The Lowry method was employed for evaluating the protein content of the samples (Daemi et al. 2019a) and MDA content was used for assessing the lipid peroxidation rate. To assess the TTM, the collected granulation tissue sample was homogenized and the supernatant was added to Tris-EDTA as reported by Daemi et al. (2019a).

### Statistical analysis

The results were reported as mean ± standard deviation and analyzed by Graph Pad Prism Software. One-way ANOVA was utilized to analyze the results. Dunnett’s test for pair-wise comparisons was used for evaluating the effect of treatments and *p* < 0.05 was considered to be of significance.

## Results

### Composition of the Zataria multiflora essential oil

**Figure 1.**
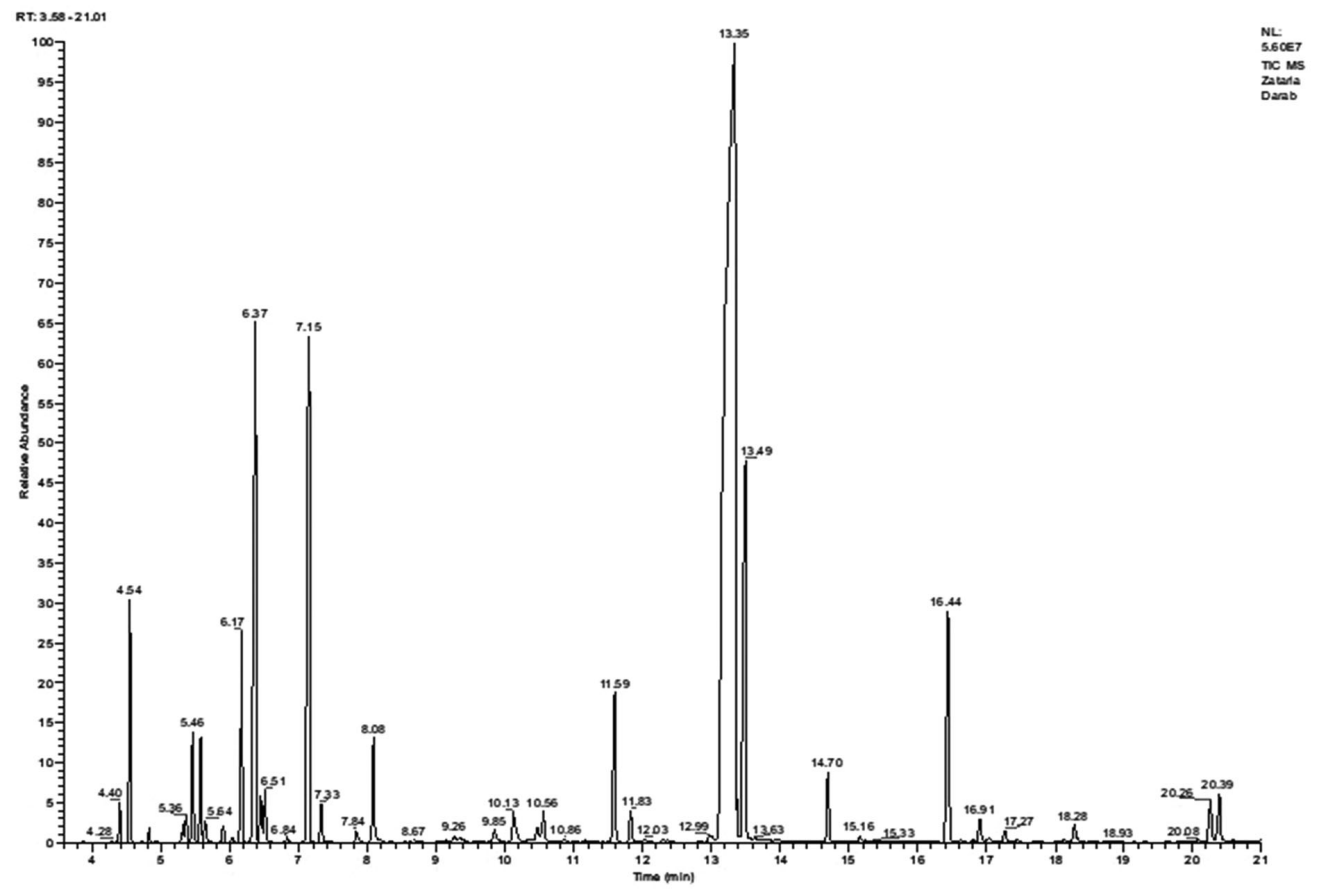
GC-MS chromatogram of the essential oil of *Zataria multiflora*.

The analysis of *Zataria multiflora* essential oil with GC-FID and GC-mass spectrometry (MS) identified 29 compounds which accounted for 99.6% of the total essential oil composition (Table 1). It contained thymol (52.90%), *p*-cymene (9.10), γ-terpinene (8.10%) and carvacrol (6.80%) as major constituents (Figure 1).

**Table 1.** Chemical constituents of *Zataria multiflora* essential oil.

No.	Compound	RI-Cal^a^	RI-Lit^b^	%
1	α-Thujene	926	924	0.4
2	α-Pinene	933	932	2.6
3	Camphene	949	946	0.1
4	β-Pinene	977	974	0.3
5	3-Octanone	982	979	1.2
6	Myrcene	988	988	1.1
7	3-Octanol	992	988	0.2
8	α-Phellandrene	1005	1002	0.2
9	α-Terpinene	1016	1014	2.5
**10**	***p*-Cymene**	1025*	1020	9.1*
11	Limonene	1028	1024	0.7
12	1,8-Cineole	1031	1026	0.6
**13**	**γ-Terpinene**	1059*	1054	8.1*
14	*cis*-Sabinene hydrate	1067	1065	0.5
15	Terpinolene	1089	1086	0.1
16	Linalool	1099	1095	1.4
17	Borneol	1170	1165	0.2
18	Terpinen-4-ol	1181	1174	0.6
19	α-Terpineol	1198	1186	0.5
20	Thymol methyl ether	1237	1232	2.1
21	Carvacrol methyl ether	1246	1241	0.4
**22**	**Thymol**	1303*	1289	52.9*
**23**	**Carvacrol**	1308*	1298	6.8*
24	Thymol acetate	1355	1349	1.0
25	(*E*)-Caryophyllene	1424	1417	3.8
26	Aromadendrene	1442	1439	0.4
27	α-Humulene	1456	1452	0.2
28	Viridiflorene	1496	1496	0.3
29	Spathulenol	1581	1577	0.6
				
	Monoterpene hydrocarbons			26.2
	Oxygenated monoterpenes			67.4
	Sesquiterpene Hydrocarbons			4.6
	Oxygenated sesquiterpenes			1.4
	Total			99.6

^a^ RI-Cal: retention indices calculated based on C_6_–C_24_ *n*-alkenes from a DB-5 column.

^b^ RI-Cal: retention indices retrieved from literature (Adams 2007).

* Bold values show major compounds in the essential oil.

### Wound area

**Figure 2.**
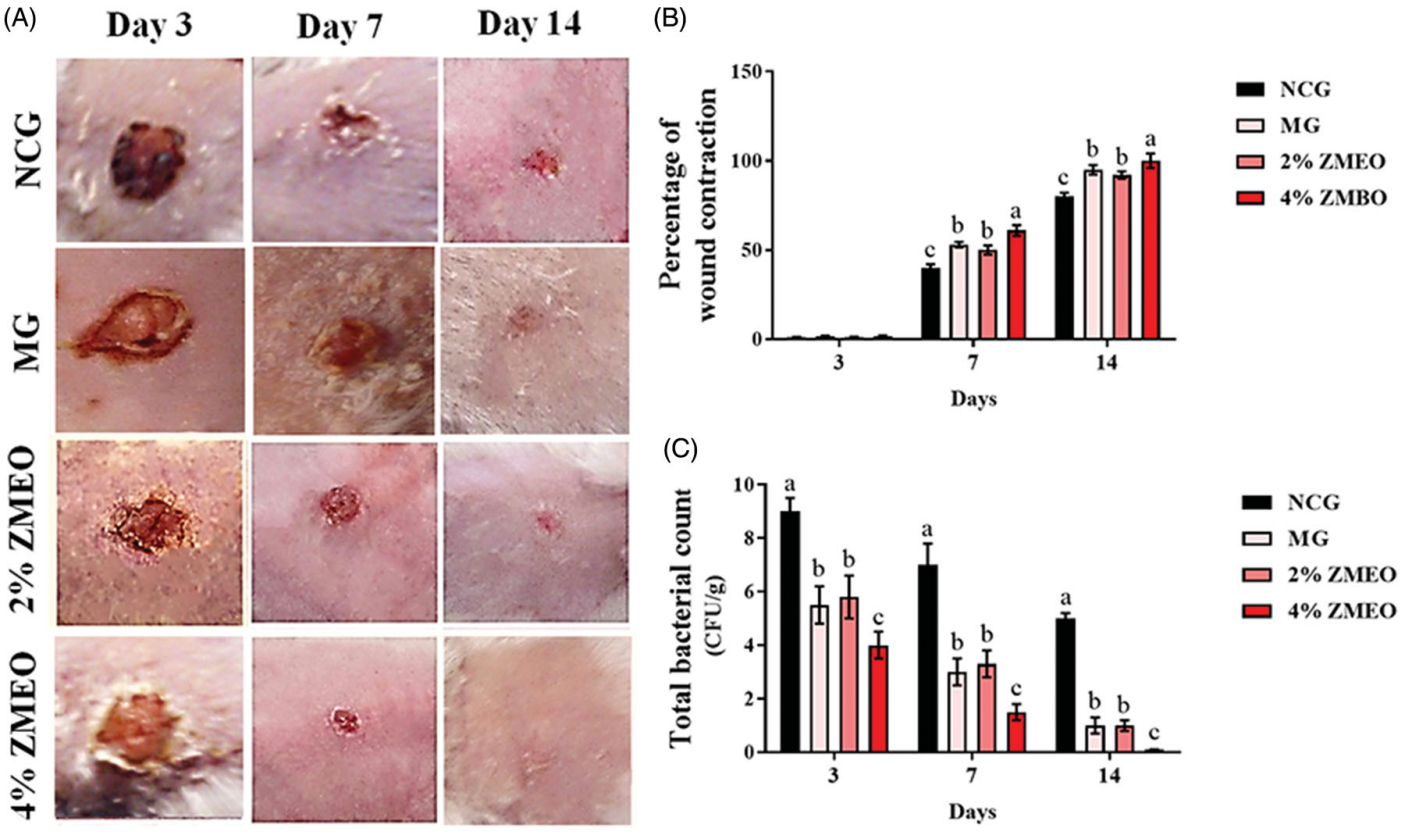
The effects of topical administration of ZMEO on (B) wound contraction rate and (C) tissue bacterial count (CFU/g) in different days. Superscripts (a–d) indicate significant differences in same day at *p* < 0.05.

The findings for the effects of ZMEO on the percentage of wound contraction are shown in Figure 2. The results implied that topical administration of therapeutic ointments significantly (*p* < 0.05) increased wound contraction rate on days 7 and 14 compared to the NCG group (Figure 2(A,B)). The comparison between treatment groups revealed that a higher wound contraction rate was observed in the 4% ZMEO-treated group compared to the 2% ZMEO-treated and MG groups (*p* < 0.05).

### The bacteriological count

The results concerning the effects of ZMEO on tissue bacteria counts are represented in Figure 2(C). The results showed that topical administration of therapeutic ointments significantly decreased bacterial colonization in all the days compared to the NCG group (*p* < 0.05). The comparison between treatment groups demonstrated that a higher level of ZMEO (4%) significantly decreased the total bacterial count compared to that of a lower level of it (2%) and MG groups (*p* < 0.05).

### Histopathological parameters

**Figure 3.**
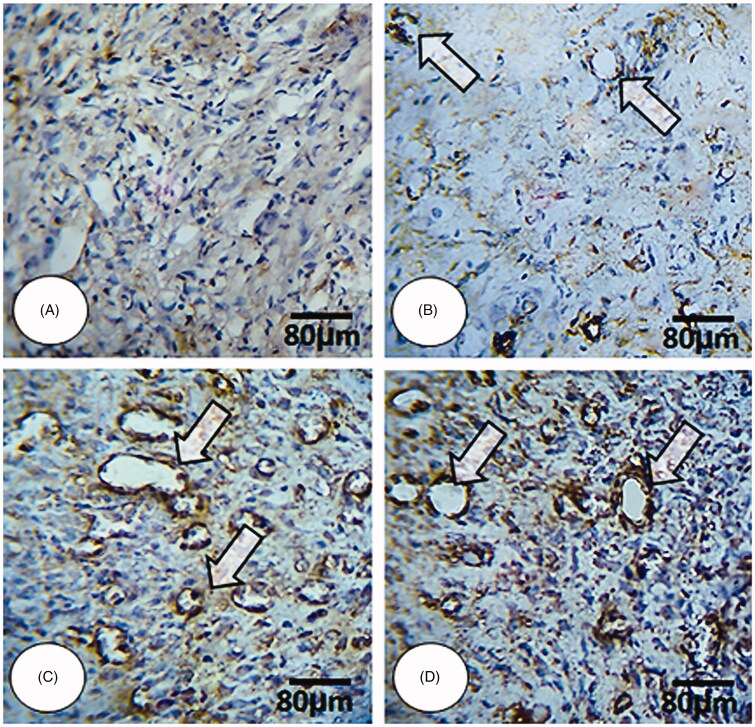
Immunohistochemical staining for angiogenesis:; (A) NCG group, (B) MG group, (C and D) 2 and 4% ZMEO-treated groups. See elevated angiogenesis in ZMEO-treated group (arrows) on day 7 after wound induction. CD 31 staining, 400×.

**Figure 4.**
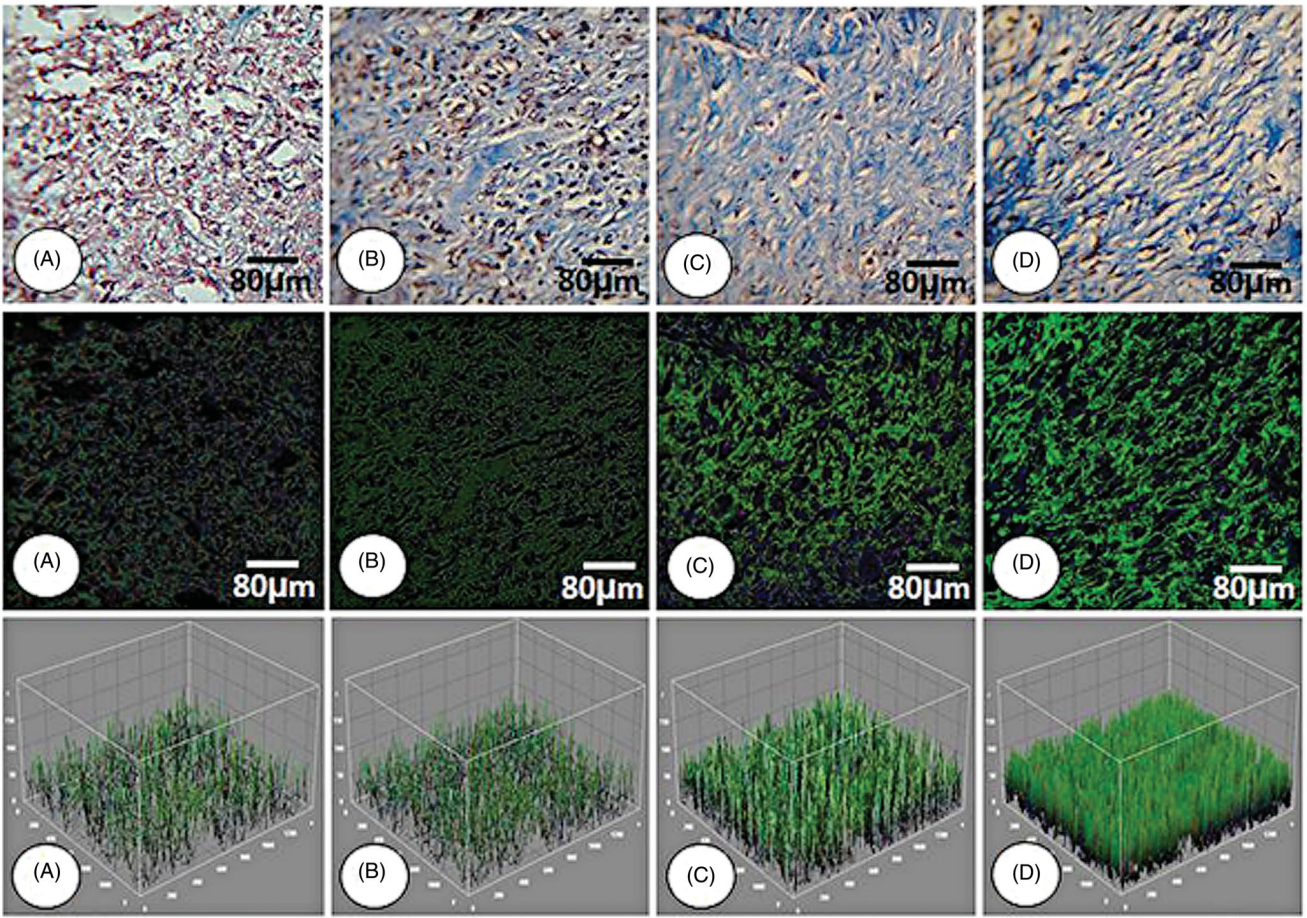
Cross section from wound area: (A) NCG group, (B) MG group, (C and D) 2 and 4% ZMEO-treated groups. Note well-formed collagen deposition in cross sections from ZMEO-treated animals on day 7 after wound induction (first row), which is significantly increased on day 14 after injury (second row). The third row represents the software analyze for collagen intensity. The cross sections from animals in ZMEO-treated groups exhibited condense collagen deposition versus NCG and MG groups. Masson-trichrome staining and fluorescent staining for collagen, 400× magnification.

**Figure 5.**
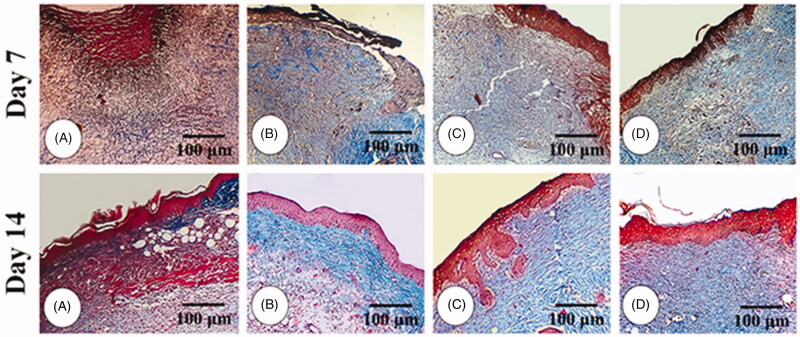
Cross section from wound area: (A) NCG group, (B) MG group, (C and D) 2 and 4% ZMEO-treated groups. See well re-epithelialization in ZMEO-treated animals. The re-epithelialization initiated on day 7 after wound induction in ZMEO-treated animals. However, the cross sections from NCG and MG groups are not representing epithelialization. Note well-organized dermis and complete epithelialization with well-formed papillae in ZMEO-treated animals in comparison to NCG and MG groups. Masson-trichrome staining, 100×.

The results for histopathological parameters are represented in Table 2. The results showed that edoema score was significantly higher in the NCG group. It was observed to be significantly lower in the animals treated with the 4% ZMEO-treated group and also in 2% ZMEO and MG-treated groups. The findings implied that the lowest edoema was observed in 4% ZMEO in day 14; meanwhile, there was no observed edoema in the 4% ZMEO group on day 14. The results showed that immune cells were significantly lower in ZMEO groups in day 3 compared to other groups (*p* < 0.05), yet it decreased on days 7 and 14 in the ZMEO groups compared to other groups (*p* < 0.05). It means that the application of the ZMEO decreases immune cells in days 7 and 14, and increased it on day 3. Angiogenesis (Figure 3) and fibroblast infiltration were significantly higher in all treated groups compared to the NCG group and the highest level was observed in level of the 4%-ZMEO group. The results for collagen condensation (Figure 4) and re-epithelialization (Figure 5) revealed that parameters were significantly higher in the ZMEO groups, which confirms the results.

**Table 2.** The effects of ZMEO on histopathological parameters in different days.

	Edoema	Immune cells	New vessels (mm^2^)	Fibroblast (mm^2^)	Collagen	Epithelium (µm)
			Day 3			
NCG	++++	125.41 ± 4.51^a^	0.25 ± 0.13^d^	0.73 ± 0.23^d^	–	–
MG	+++	109.11 ± 7.24^b^	1.14 ± 0.21^c^	1.58 ± 0.41^c^	–	8.71 ± 1.11^c^
2% ZMEO	+++	82.21 ± 7.31^c^	1.81 ± 0.11^b^	2.35 ± 0.57^b^	+	13.21 ± 1.21^b^
4% ZMEO	++	63.12 ± 7.51^d^	2.30 ± 0.21^a^	3.50 ± 0.21^a^	++	25.41 ± 1.71^a^
			Day 7			
NCG	+++	93.12 ± 5.41^a^	3.41 ± 0.51^d^	1.47 ± 0.33^d^	+	14.21 ± 3.41^d^
MG	++	72.21 ± 4.14^b^	8.21 ± 0.74^c^	3.92 ± 0.32^c^	++	31.24 ± 4.12^c^
2% ZMEO	++	65.10 ± 3.13^c^	11.24 ± 0.91^b^	4.45 ± 0.32^b^	++	65.12 ± 3.74^b^
4% ZMEO	++	55.10 ± 4.11^d^	14.51 ± 0.51^a^	7.81 ± 0.42^a^	+++	89.12 ± 3.41^a^
			Day 14			
NCG	+	76.41 ± 3.24^a^	3.31 ± 0.51^b^	2.23 ± 0.73^d^	++	18.51 ± 2.12^d^
MG	–	63.11 ± 3.21^b^	5.21 ± 1.31^a^	4.75 ± 0.23^c^	+++	48.51 ± 3.51^c^
2% ZMEO	–	41.24 ± 4.21^c^	5.81 ± 1.31^a^	7.51 ± 0.91^b^	+++	99.41 ± 2.13^b^
4% ZMEO	–	35.80 ± 7.10^d^	5.41 ± 1.51^a^	11.32 ± 0.91^a^	++++	131.51 ± 8.41^a^

Superscripts (a–d) indicate significant differences in same day at *p* < 0.05.

### Molecular analyses

**Figure 6.**
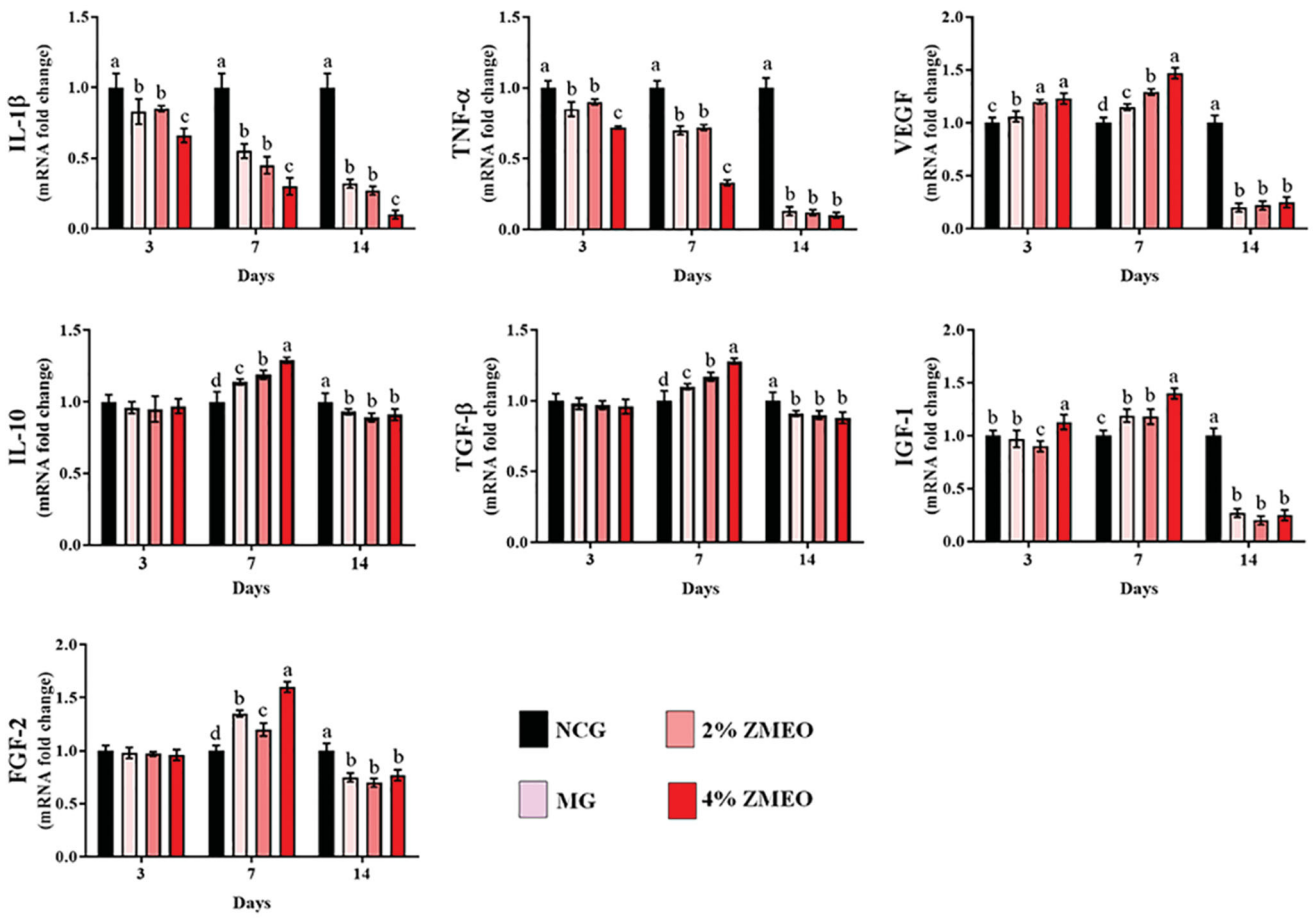
The effects of topical application of ZMEO on gene expression. Superscripts (a–d) indicate significant differences in same day at *p* < 0.05.

The results for gene expression are presented in Figure 6. Gene expression of IL-1β and TNF-α was significantly (*p* < 0.05) lower on all sampling days at treated groups compared to the NCG group. The greatest decrease was observed in the 4% ZMEO-treated animals. Gene expressions of IGF-1 and VEGF was significantly (*p* < 0.05) higher at all treated groups on day 7 after the wounding, but the levels significantly decreased on day 14. The expression of IL-10 level increased on day 7 whereas it decreased on day 14 compared to the NCG group (*p* < 0.05). Further analysis of gene expressions of TGF-β and FGF-2 indicate that its level does not have significant difference (*p* > 0.05) on day 3, yet its level significantly (*p* < 0.05) increased in all treated groups, particularly at 4% ZMEO-treated animals on day 7. The amount of TGF-β and FGF-2 gene expression levels also were significantly (*p* < 0.05) reduced on day 14 compared to the NCG group.

### Antioxidant status

**Figure 7.**
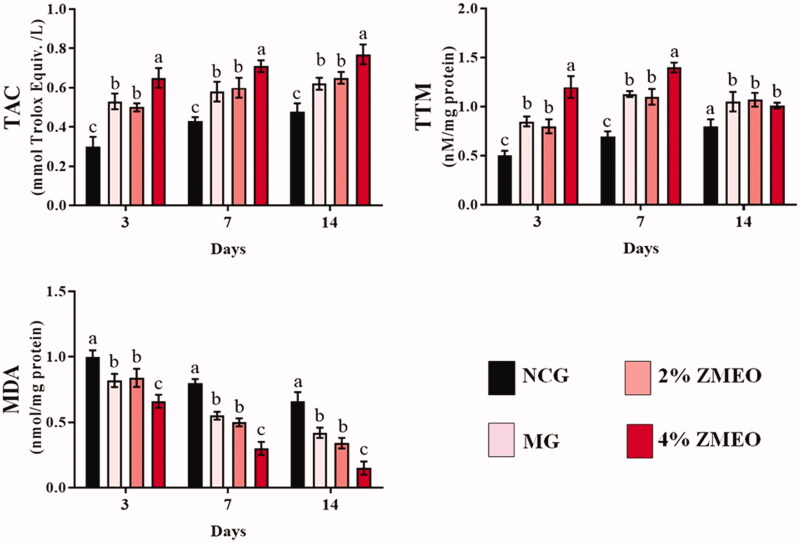
The effects of topical application of ZMEO on antioxidant properties. Superscripts (a–d) indicate significant differences in same day at *p* < 0.05.

Antioxidant status in different groups is represented in Figure 7. Using the 2% and 4%-ZMEO significantly (*p* < 0.05) increased TAC and TTP levels compared to the NCG group in all of the days after the wounding. Interestingly, the highest increase was observed in the 4% ZMEO-treated animals. Further analysis of MDA level indicates significant (*p* < 0.05) decrease in all of the treated groups compared to the NCG group in all of the days after the wounding. The highest decrease was observed in the 4% ZMEO-treated group.

## Discussion

Wounds infected by pathogenic bacteria such as *S. aureus*, and *P. aeruginosa* are believed to be serious challenges for regarding their management and treatment (Manzuoerh et al. 2019). Different agents are employed for the treatment of wounds. Active compounds of the essential oils can have pivotal roles in their treatment. The findings indicated that thymol (52.90%), *p*-cymene (9.10), γ-terpinene (8.10%) and carvacrol (6.80%) were the major constituents in the essential oil. Similar to our results, previous studies demonstrated that the major compounds of *Z. multiflora* essential oil were thymol, carvacrol and p-cymene (Aida et al. 2015), thymol, c-terpinene, p-cymene, and carvacrol (Saei-Dehkordi et al. 2010), carvacrol (Misaghi and Basti 2007), thymol, carvacrol, and p-cymene (Sharififar et al. 2007). The results showed that ZMEO accelerated wound healing compared to the NCG group and synthetic agent of mupirocin. Herein, the results are described based on the mechanisms and active compounds, step-by-step.

Inflammation is the first step in the wound healing process, in which different factors and genes are involved. Inflammation is triggered once the injury induces to living tissues by live organisms, such as bacteria and/or physical injury and faulted immune response. Inflammation removes foreign substances in order to improve the wound healing process (Garrett et al. 2010). It promotes macrophages, neutrophils and immune cells for initiation of inflammation (Oguntibeju 2018). IL-1β and TNF-α are commonly produced by immune cells, such as macrophages and mast cells (Thacker et al. 2007). The results showed that the topical application of ZMEO decreased immune cells and the expressions of IL-1β and TNF-*α* in the first 3 days. The results implied a positive relation between the number of immune cells with the expression of IL-1β and TNF-*α* in the treatments. Meaning that immune cells increase the expression of IL-1β and TNF-*α* in the first 3 days. Increased inflammation delays the wound healing process. The findings revealed that topical administration of ZMEO decreased inflammation on days 7 and 14, and acted as an antibacterial and anti-inflammation agent. Antibacterial and anti-inflammatory activity of ZMEO is attributed to its compounds. The essence contains thymol and carvacrol that are of antibacterial and anti-inflammatory properties. It was reported that the administration of carvacrol significantly reduced TNF-α levels in pleural lavage (Guimarães et al. 2012). The anti-inflammatory activity of thymol leads to prevention of the phosphorylation of extracellular signal-regulated protein kinase, c-Jun N-terminal kinase, and nuclear factor-κB (Liang et al. 2014). Carvacrol prevented leukocyte migration, and decreased edoema, and showed anti-inflammatory effects (Fachini-Queiroz et al. 2012). The results indicated that immune cells decreased and expression of IL-1β and TNF-*α* also decreased in ZMEO groups. The findings showed that the total bacterial count significantly decreased in ZMEO groups compared to the NCG group. Mupirocin is used as a bactericide agent for the treatment of infected wounds. The results convey that ZMEO, particularly at a higher level, have better antibacterial activity compared to mupirocin. Higher total bacterial count increases the inflammation and delays the wound healing process. It means that topical administration of ZMEO decreases the inflammation with its antibacterial activity. It was reported that the antibacterial activity of *Zataria multiflora* is in view of its compounds (Shafiee et al. 1999; Barkhori-Mehni et al. 2017). In essence, ZMEO decreased the inflammation and inflammatory-associated parameters. Higher levels of ZMEO provide higher doses of active compounds that act as anti-inflammatory factors. Decreased inflammation leads the wound healing process towards the proliferative phase.

Our findings implied that the topical application of ZMEO improved the proliferative phase. The results showed that the genes expression of VEGF, FGF-2 and IGF-1 increased in ZMEO groups. IGF-1 increases the transport of glucose during the wound healing process and improves the proliferative phase (Daemi et al. 2019b). VEGF induces angiogenesis and stimulates cell migration and proliferation (Norton & Popel 2016; Farahpour et al. 2020; Gharaboghaz et al. 2020). FGF-2, a protein that participates in the wound healing process (Souto et al. 2020), together with other growth factors, such as VEGF, are reported to increase the angiogenesis and indirectly support the cellular nutrient, oxygen and energy supplements (Harding et al. 2002). So far, studies have not investigated the effect of ZMEO on genes expression of VEGF, FGF-2 and IGF-1. It means that VEGF improves angiogenesis, and vessel number was significantly higher in ZMEO groups. VEGF improved angiogenesis and number of vessels. Other genes involved were TGF-β, and IL-10 whose expressions increased on day 7. IL-10 is a factor that reduces the production of pro-inflammatory cytokine, but TGF-β leads the phase towards the proliferative phase by improving the proliferation and differentiation of fibroblasts, collagen production, and wound contraction (Khezri et al. 2020). Seemingly, both genes are of greatly important roles in decreasing the inflammation and passing to the proliferative phase. The IHC results indicated improved collagen deposition. The biosynthesis of collagen during the proliferation stage has pivotal roles in dermal maturation in which some factors are involved (Yakaew et al. 2016; Daemi et al. 2019a). The results showed that the topical application of ZMEO increased fibroblast, which is due to its effect on IGF-1. It was reported that IGF-1 increases fibroblast cells in *in vitro* assessment (Lisa et al. 2011). Improving cellular proliferation and differentiation are essential for shortening the healing time (Oryan et al. 2015; Karimzadeh and Farahpour 2017). Proliferative effect of ZMEO is partly owing to its antioxidant activity. The topical application of ZMEO improved antioxidant activity. Previous studies have reported antioxidant activity of *Zataria multiflora* essential oil (Fatemi et al. 2012; Karimian et al. 2012; Kavoosi and Teixeira da Silva 2012). Oxidative stress in the wound area increases the damage of proteins, nucleotides and lipid levels (Cano Sanchez et al. 2018; Farahpour et al. 2020; Gharaboghaz et al. 2020) and decreases the antioxidant activity. This fact is associated with increased changes in the antioxidant profile, which are contributed to the increase in the levels of MDA content (Sharma et al. 2012; Bardaa et al. 2016; Vittorazzi et al. 2016).

## Conclusion

The topical application of ZMEO decreased the inflammation and the total bacterial count, which progresses the wound healing process towards the proliferative phase. It increases the expression of proliferative genes, helps to alleviate the inflammation phase and accelerates the proliferative phase. Antioxidant activity of ZMEO accelerated the wound healing process. All the above-mentioned factors hasten the wound healing process, as seen for the wound area. Accordingly, applying ZMEO only and/or in combination with chemical agents for the treatment of wound healing could be suggested.
